# A Policy-Based Intervention for Physical Activity and Screen Time in Childcare Outside School Hours: A Cluster Randomized Clinical Trial

**DOI:** 10.1001/jamanetworkopen.2026.36741

**Published:** 2026-10-05

**Authors:** Carol Maher, Luke Wolfenden, Nicole Nathan, Jacinta Brinsley, Ty Ferguson, Rachel Curtis, Rosa Virgara, Alyce Barnes, Svetlana Bogomolova, Kylie Brannelly, Dylan P. Cliff, Perry Campbell, Adrian Esterman, Lucy K. Lewis, Rachel K. Milte, Michael Noetel, Anthony D. Okely, Mandy Richardson, Richard R. Rosenkranz, Natasha Schranz, Rebecca M. Stanley, R. Glenn Weaver, Hayley Christian

**Affiliations:** 1 Alliance for Research in Exercise, Nutrition and Activity (ARENA), Adelaide University, South Australia, Australia; 2 School of Medicine and Public Health, The University of Newcastle, Newcastle, New South Wales, Australia; 3 National Centre of Implementation Science, The University of Newcastle, Newcastle, New South Wales, Australia; 4 Centre for Social Impact, Flinders Business School, Flinders University, Adelaide, South Australia, Australia; 5 Queensland Children’s Activities Network, Woodend, Queensland, Australia; 6 Early Start, School of Education, University of Wollongong, Wollongong, New South Wales, Australia; 7 Australian Children’s Education and Care Quality Authority (ACECQA), Darlinghurst, New South Wales, Australia; 8 Caring Futures Institute, Flinders University, Adelaide, South Australia, Australia; 9 School of Psychology, The University of Queensland, Brisbane, Queensland, Australia; 10 School of Medical, Indigenous and Health Sciences, University of Wollongong, Wollongong, New South Wales, Australia; 11 Department for Education, Adelaide, South Australia, Australia; 12 Department of Kinesiology and Nutrition Sciences, University of Nevada, Las Vegas; 13 Preventive Health SA, Adelaide, South Australia, Australia; 14 School of Social Sciences, Faculty of the Arts, Social Sciences and Humanities, University of Wollongong, Wollongong, New South Wales, Australia; 15 Department of Exercise Science, Arnold School of Public Health, University of South Carolina, Columbia; 16 The Kids Research Institute Australia, The University of Western Australia, Perth, Western Australia, Australia; 17 School of Population and Global Health, The University of Western Australia, Perth, Western Australia, Australia

## Abstract

**Importance:**

Extended education programs reach tens of millions of children globally during discretionary hours when physical activity competes with screen use, yet scalable strategies to improve movement behaviors are limited.

**Objective:**

To evaluate the effectiveness of Activated Outside School Hours Care (OSHC), a policy-based intervention to improve physical activity and screen time practices in extended education settings.

**Design, Setting, and Participants:**

This cluster randomized clinical trial was conducted in Australia’s system of organized before- and after-school care in 3 Australian states (South Australia, New South Wales, and Western Australia) between August 1, 2022, and December 31, 2024, with assessments at baseline, 3 months, and 12 months.

**Intervention:**

Activated OSHC is a web-based intervention offering voluntary accreditation to services aligning their policies with national physical activity and screen time guidelines through staff training. Services were randomized 1:1 to intervention or usual practice, in which services continued their typical programming.

**Main Outcomes and Measures:**

The primary outcome was service-level adherence to OSHC sector physical activity and screen time guidelines. Secondary outcomes included staff physical activity–enabling behaviors, children’s moderate-to-vigorous physical activity and recreational screen time, and availability of physical activity equipment, assessed through direct observation. Intention-to-treat and per-protocol analyses were performed.

**Results:**

Of 197 services (101 in the intervention group and 96 in the control group), 182 (92%; 90 in the intervention group and 92 in the control group) completed the 12-month follow-up. Of the 101 services in the intervention group, 48 (48%) achieved accreditation within 12 months. Intention-to-treat analyses showed no significant effects for guideline adherence or children’s activity levels. However, the services in the intervention group demonstrated greater staff physical activity–promoting behaviors during before-school care (odds ratio [OR], 1.72; 95% CI, 1.27-2.33) and greater availability of physical activity equipment during before- (OR, 1.21; 95% CI, 1.00-1.46) and after-school care (OR, 1.16; 95% CI, 1.04-1.30). In per-protocol analyses, accredited services were more likely than controls to meet all guidelines (OR, 7.35; 95% CI, 1.39-38.86), with improved after-school screen time adherence (OR, 5.63; 95% CI, 1.16-27.22). In per-protocol analyses, accredited services showed greater staff physical activity–enabling behaviors (OR, 3.00; 95% CI, 2.10-4.28), and children engaged in more physical activity during before-school care (incidence rate ratio, 1.23; 95% CI, 1.05-1.43) compared with controls.

**Conclusions and Relevance:**

This randomized clinical trial found few population-level changes in intention-to-treat analyses. However, services achieving accreditation demonstrated consistent improvements in guideline adherence, staff behaviors relating to physical activity, and children’s physical activity. The intervention was effective when implemented, but limited uptake constrained population-level impact. The accreditation model is viable for practice improvement, but policy- or system-level strategies may be needed to drive widespread adoption.

**Trial Registration:**

Australian New Zealand Clinical Trials Registry Identifier: ACTRN12622000393752

## Key Points

**Question:**

Can a policy-based intervention improve physical activity and screen time practices in organized care programs outside the school day?

**Findings:**

In this cluster randomized effectiveness–implementation trial of 197 Australian Outside School Hours Care services, 47.5% of intervention services achieved accreditation. At 12 months, accredited services were 7 times more likely to meet national physical activity and screen time guidelines than control services.

**Meaning:**

Findings of this trial indicate that a policy-based intervention can meaningfully improve service practices, staff behaviors, and children’s physical activity, but achieving impact at scale depends on service uptake and engagement.

## Teaser

This cluster randomized clinical trial assesses the effectiveness of a policy-based intervention to improve physical activity and screen time practices in programs in Australia offering childcare outside of regular school hours.

## Introduction

Insufficient physical activity and excessive recreational screen time affect children globally and are associated with adverse physical, mental, and social health outcomes.^1,2^ Despite longstanding guidelines, most school-aged children fail to meet recommended movement targets,^3,4^ particularly during discretionary hours outside the school day.^5^ School-based interventions have shown mixed results, constrained by curriculum pressures and competing demands,^6,7^ highlighting the need for complementary population health strategies that can reach large numbers of children beyond the formal school day.^8^

Globally, tens of millions of children attend organized care programs outside school hours, known as after-school programs in the US, school-age childcare across Europe, and outside school hours care (OSHC) in Australia.^9,10^ In Australia, approximately one-fourth of primary school-aged children attend OSHC each week,^11,12^ providing them with regular access to physical activity during a period when sedentary recreation competes with physical activity.^13^ Australia’s OSHC sector has codeveloped service-level physical activity and screen time guidelines^14^ that specify modifiable practices relating to scheduled activity and screen exposure.

Building on a decade of partnership with the OSHC sector, the Activated OSHC program was codesigned to support services to implement national physical activity and screen time guidelines through a systems-based approach targeting staff training, policy development, and environmental supports. Rather than targeting individual children or families, the intervention aims to embed guideline-consistent practices into service structures, policies and workforce capacity. This intervention responds to calls for stronger evidence on scaling public health interventions^15^ and is the first large-scale randomized clinical trial (RCT), to our knowledge, to evaluate implementation of national physical activity and screen time guidelines in OSHC services.^16^ This study evaluated whether a policy-focused intervention improved service-level practices, staff behaviors, OSHC environments, and children’s observed physical activity and recreational screen time under usual conditions. The findings inform whether voluntary accreditation can strengthen movement-supportive environments in organized care settings.

## Methods

### Study Design

This type 2 hybrid effectiveness–implementation cluster RCT was conducted from August 1, 2022, to December 31, 2024, with assessments at baseline, 3 months, and 12 months. Type 2 hybrid trials assess both effectiveness and implementation outcomes; this article reports effectiveness outcomes alongside intervention uptake and accreditation. Services were randomized 1:1, with service as the unit of randomization and analysis. The trial was prospectively registered; approved by ethics committees at the University of South Australia, University of Western Australia, and University of Newcastle; and approved by relevant education sector bodies (Catholic Education South Australia, Department of Education South Australia, and Department of Education Western Australia). OSHC coordinators provided written informed consent. This RCT was conducted according to Consolidated Standards of Reporting Trials (CONSORT) reporting guideline^17^ and described using the TIDieR checklist.^18^ The approved protocol is available in Supplement 1 and a detailed version has been published elsewhere.^15^

### Participants

OSHC services were recruited from South Australia, New South Wales, and Western Australia. Eligible services provided both before-school and after-school care, enrolled 10 or more children daily, had internet access, and were led by a coordinator able to read, speak, and write in English.

Services within approximately 120 km of Adelaide, Newcastle, and Perth were identified using Australian Children’s Education and Care Quality Authority registers. Metropolitan services were stratified by socioeconomic status using the Socio-Economic Indexes for Areas Index of Community Socio-Educational Advantage,^19^ with recruitment across low, medium, and highsocioeconomic tertiles (n = 167). Thirty regional services were recruited using convenience sampling (approximately 10 per state).

Recruitment occurred between August 2022 and December 2023. Coordinators were contacted by email and then telephone to confirm eligibility and interest, and provided written informed consent. Parents and guardians were informed and could opt out of child observation. Services completing 12-month follow-up received AUD $250 (approximately US $180).

### Intervention and Control Conditions

Intervention services received access to Activated OSHC, a multicomponent, web-based, policy-focused intervention developed through 5 online codesign workshops with 21 OSHC educators and coordinators, with iterative sector partner input on educational scripts, activities, resources, and implementation materials. The intervention supports services to align policies and practices with Australian OSHC physical activity and screen time guidelines^14^ through staff training, policy development, and implementation resources. Sector-identified barriers and enablers were mapped to the Theoretical Domains Framework^20^ by 2 investigators (C.M. and R.V.), then reviewed with implementation science experts (L.W. and N.N.) to specify the behavioral targets of the intervention and inform the coordinator survey.

Services accessed an online manual covering active play scheduling and screen time management. Staff completed 4 online training modules: rationale for change, strategies for engaging physical activity, reducing and “savoring” screen time, and accreditation (eFigure 1 in Supplement 2). Additional resources included posters, family communication materials, and accreditation signage (eFigure 2 in Supplement 2).

To achieve accreditation, services submitted their physical activity and screen time policy for assessment against OSHC guidelines for before- and after-school scheduling, and required 50% or more of staff to complete online training. Initial accreditation was valid for 3 years and assessed and granted by the central research site at Adelaide University, formerly the University of South Australia.

Control services continued usual programming. The Activated OSHC website was password protected and restricted to services in the intervention group. Control group services were offered program access after 12-month assessment.

### Outcomes and Measures

All outcomes were assessed at baseline, 3 months, and 12 months. The primary outcome was service-level adherence to the Australian OSHC physical activity and screen time guidelines, as determined from coordinators’ responses to the OSHC Schedule Tool. Adherence was defined as meeting all 4 components: 45 minutes or more of physical activity and 30 minutes or less of recreational screen time before school and 90 minutes or more of physical activity and 60 minutes or less of recreational screen time after school. At each assessment, coordinators completed the OSHC Schedule Tool, a validated instrument capturing types and duration of activities scheduled during a typical day in 15-minute increments.^21^ For before- and after-school care, coordinators indicated which activities were typically offered from a range of general (eg, arts and crafts, board games), screen time (eg, TV, video games), and physical activities (eg, access to an outdoor playground, staff-led active games), and the time each activity was typically offered by selecting all relevant 15-minute increments.

Secondary outcomes included children’s observed physical activity and screen time during OSHC sessions, staff physical activity–related behaviors, and availability of screens and physical activity equipment, measured using the System for Observing Play and Leisure Activity in Youth (SOPLAY)^22^ and System for Observing Staff Promotion of Activity and Nutrition (SOSPAN),^23^ during 1 before-school and 1 after-school session at each time point. Observation visits occurred on an unannounced day within a 2-week window and were conducted by trained research staff blinded to group allocation. SOPLAY involved systematic scans of predefined activity zones recording the number of children (count data) engaged in sedentary physical activity, light physical activity, and moderate-to-vigorous physical activity (MVPA), and recreational screen time and the conditions of each play zone (eg, whether it had physical activity equipment present). An additional item was added post hoc to capture availability of screens at each scan (yes or no). After each SOPLAY scan, a second scan was performed documenting staff behaviors (recorded as a binary indicator of observed or not observed) in relation to physical activity (categorized as either engaged, instructing, promoting [enabling behaviors such as giving verbal praise for participation in physical activity, participating in physical activity with children, or leading physically active games], discouraging [eg, telling children to stop engaging in a physical activity], withholding [eg, removing a child from physical activity], punishing [eg, running laps; disabling behaviors] or off-task).

Prior to data collection, all research assistants completed training to ensure standardized use of SOPLAY and SOSPAN. This training comprised independent reading of 5 peer-reviewed articles covering the conceptual basis and operationalization of each tool, followed by a full-day in-person training session incorporating 11 instructional videos on tool operationalization and study procedures. Research assistants then completed a supervised observation session at an active OSHC service, scanning independently alongside an experienced researcher. Research assistants were required to meet an agreement threshold within 5% before collecting data, following published protocols.^24^

### Sample Size

Sample size was determined based on the primary outcome. We estimated the intervention would increase the proportion of services meeting guidelines by 20 percentage points, from 41% to 61%, informed by a national survey of approximately 570 OSHC services.^21^

Assuming 80% power, a 2-sided α = .05, and 3 repeated measurements, a minimum of 75 services per group was required. To allow for attrition, the recruitment target was 192 services. No interim analyses or formal stopping rules were applied.

### Randomization and Blinding

After baseline data collection, services were randomly allocated 1:1 to intervention or control using a computer-generated allocation sequence within REDCap, with block randomization within strata. Stratified randomization ensured balance by state and location (metropolitan or regional), with metropolitan services further stratified by socioeconomic tertile. Randomization occurred after baseline assessments, ensuring allocation concealment.

Due to the nature of the intervention, coordinators and staff could not be blinded. However, research staff conducting outcome assessments were blinded throughout. To minimize contamination, website access was password protected and restricted to services in the intervention group.

### Statistical Analysis

Analyses were conducted according to intention-to-treat (ITT) principles, with services analyzed in originally randomized groups regardless of intervention uptake. Per-protocol analyses compared services that complied with the study protocol and therefore met accreditation requirements with services in the control group, using the same statistical methods as the ITT analyses.

For the primary outcome, changes in the proportion of services meeting guidelines were analyzed using mixed-effects logistic regression with fixed effects for group, time, and group-by-time interaction, plus a random intercept for service.

Secondary outcomes for children’s physical activity and screen time were analyzed using mixed-effects negative binomial regression with robust SEs due to overdispersion in the count data. Staff physical activity–enabling behaviors were analyzed using mixed-effects logistic regression. Post hoc analyses of screen and physical activity equipment availability were conducted using mixed-effects logistic regression.

All models were adjusted for stratification variables (state and socioeconomic tertile for metropolitan services). Results are presented as odds ratios (ORs) or incidence rate ratios (IRRs) with 95% CIs. Statistical significance was set at 2-sided *P* < .05. Mixed-effects modeling allowed inclusion of all available data under a missing-at-random assumption. Analyses were conducted using Stata, version 18 (Stata Corp).

## Results

Between August 2022 and December 2023, 579 OSHC services were invited, 266 expressed interest and were assessed for eligibility, and 197 were randomized after baseline visits (Figure): 101 services were assigned to the intervention group and 96 to the control group. The 197 randomized services cared for approximately 10 000 children (exact number of children not available because OSHC services were enrolled and children were not enrolled).

**Figure.**
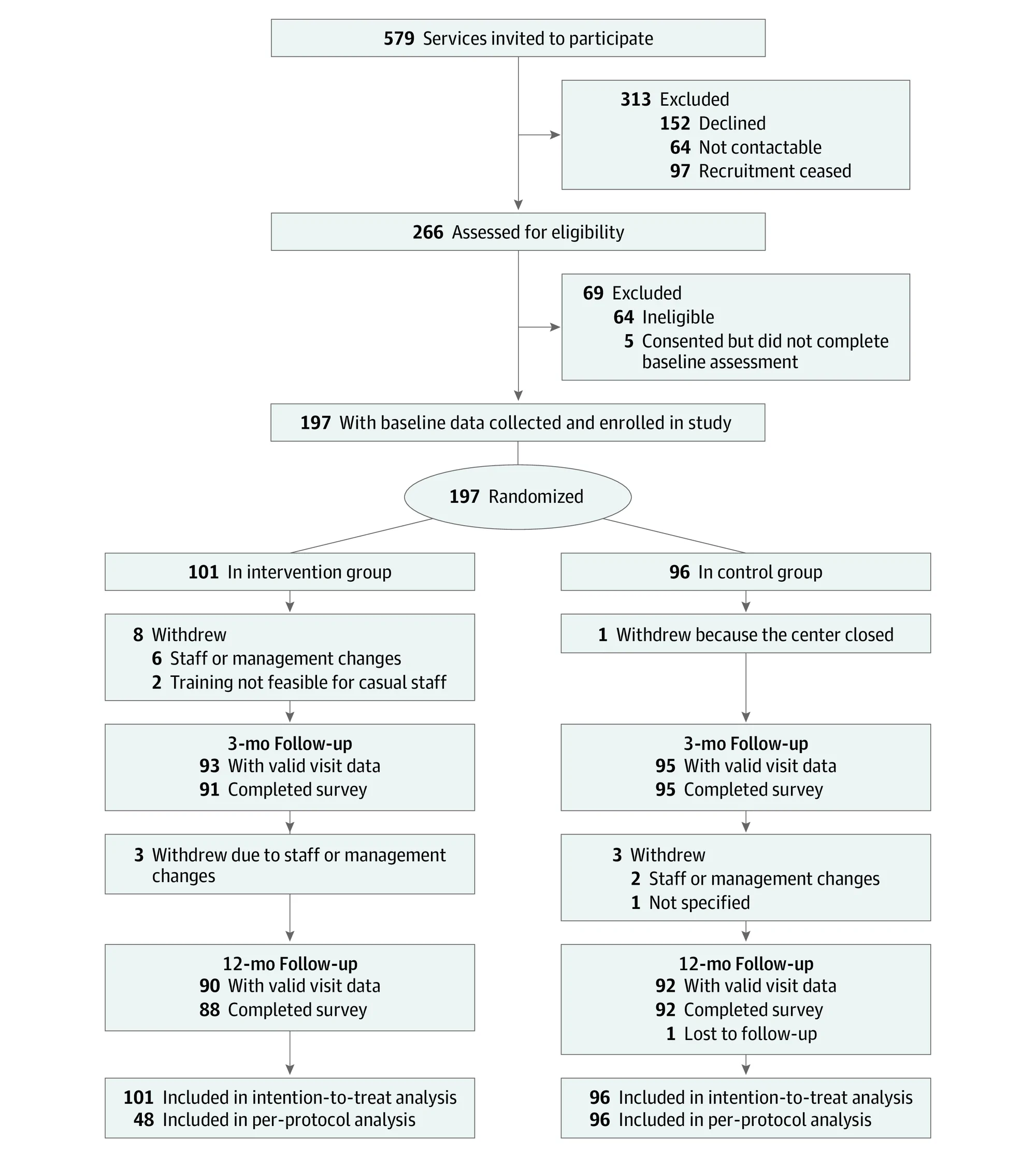
CONSORT Flow Diagram Flow of participants through the study. CONSORT indicates Consolidated Standards of Reporting Trials.

Recruitment in New South Wales was delayed by COVID-19 restrictions and industrial action, limiting recruitment there to Catholic and independent school–based services; additional services were therefore recruited in South Australia and Western Australia to meet the sample target. At 3-month follow-up, 9 services had withdrawn: 8 intervention and 1 control. A further 6 services withdrew before 12 months: 3 intervention and 3 control. Reasons for withdrawal are detailed in the Figure. At 12 months, 182 services (92%; 90 services in the intervention group and 92 in the control group) completed assessment. All randomized services were included in ITT analyses.

### Baseline Characteristics

Baseline characteristics were comparable between intervention and control groups (Table 1). Services had a median daily attendance of 46 children (IQR, 30-62 children) and 6.5 staff (IQR, 4-10 staff). Services were distributed across socioeconomic tertiles (76 low [39%], 64 middle [32%], and 57 high [29%]) and 29 (15%) were in regional areas.

**Table 1.** Baseline Characteristics of OSHC Services

Characteristic	No. (%)	
	Intervention	Control
Services		
All	101 (100)	96 (100)
New South Wales	13 (13)	11 (11)
South Australia	48 (48)	47 (49)
Western Australia	40 (40)	38 (40)
Location		
Metropolitan	85 (83)	83 (87)
Regional	16 (16)	13 (14)
SES tertile		
High	29 (29)	28 (29)
Middle	30 (30)	34 (35)
Low	42 (42)	34 (35)
Service governance		
Third-party provider	62 (61)	54 (56)
Run by school	39 (39)	42 (44)
Service size, median (IQR), No.		
Daily attendance	45 (30-60)	47.5 (27-62.5)
Staff count	6 (4-11.2)	7 (3.2-10)
**Screen time scheduling, median (IQR), min**		
Before-school session	0 (0-45)	0 (0-45)
After-school session	45 (0-90)	30 (0-90)
**Physical activity scheduling, median (IQR), min**		
Before-school session	60 (30-75)	60 (30-60)
After-school session	120 (90-150)	135 (105-150)
**Meeting screen time guidelines**		
Before-school session	70 (73)	66 (70)
After-school session	58 (60)	61 (65)
**Meeting physical activity guidelines**		
Before-school session	68 (71)	68 (72)
After-school session	77 (80)	85 (90)

Abbreviations: OSHC, Outside School Hours Care; SES, socioeconomic status.

Among intervention services, accredited services were more likely than unaccredited services to be metropolitan (47 of 48 [98%] vs 43 of 53 [81%]; *P* = .007), school-run (27 of 48 [56%] vs 12 of 53 [23%]; *P* < .001), and larger in terms of daily attendance (median, 50 [IQR, 30-68] vs 35 [IQR, 30-50]; *P* = .04) and staff count (median, 8 [IQR, 6-12] vs 5 [IQR, 3-10]; *P* = .02) (eTable 1 in Supplement 2). State and SES tertile did not differ significantly between accredited and unaccredited services.

### Program Uptake and Accreditation

Of 101 services in the intervention group, 81 (80%) registered on the Activated OSHC website. Across registered services, 538 staff signed up and 423 (79%) completed all training modules; 50 services met the training requirement of 50% or more of staff completion.

Fifty-nine services submitted a physical activity and screen time policy for review, of which 50 were approved as guideline aligned. Overall, 48 services in the intervention group (48%) met both training and policy requirements, achieved accreditation within 12 months, and comprised the per-protocol sample. Median time from randomization to accreditation was 136 days (IQR, 106-190 days).

### Primary Outcome: Service-Level Adherence to OSHC Sector Physical Activity and Screen Time Guidelines

In ITT analyses, the proportion of services meeting all guidelines did not differ significantly between groups at 3 or 12 months (Table 2). In per-protocol analyses, accredited services in the intervention group were more likely than those in the control group to meet all 4 guidelines at 12 months (OR, 7.35; 95% CI, 1.39-38.86; *P* = .02), but not at 3 months (Table 2; eFigure 3 in Supplement 2).

**Table 2.** Proportion of OSHC Services Meeting Physical Activity and Screen Time Guidelines at Baseline, 3 Months, and 12 Months

Guideline	No./total No. (%)		ARD, %	Group-by-time interaction baseline to 3 mo		No./total No. (%)	ARD, %	Group-by-time interaction baseline to 12 mo	
	Baseline	3 mo		OR (95% CI)	*P* value	12 mo		OR (95% CI)	*P* value
**All guidelines**									
Intervention (ITT)	29/101 (29)	32/91 (35)	6.0	1.56 (0.51 to 4.74)	.43	38/88 (43)	7.4	1.63 (0.53 to 5.00)	.39
Intervention (PP)	11/48 (23)	14/48 (29)	5.8	1.95 (0.41 to 9.38)	.40	24/48 (50)	20.0	7.35 (1.39 to 38.86)	.02
Control	37/96 (39)	37/95 (39)	NA	NA	NA	42/92 (46)	NA	NA	NA
**Screen time guidelines**									
Before school									
Intervention (ITT)	70/96 (73)	60/83 (72)	−2.9	0.76 (0.21 to 2.77	.68	62/86 (72)	−2.4	0.74 (0.21 to 2.67)	.65
Intervention (PP)	34/48 (71)	33/48 (69)	−4.4	0.54 (0.11 to 2.63)	.45	NA	0.6	1.07 (0.22 to 5.22)	.93
Control	66/94 (70)	66/91 (73)	NA	NA	NA	66/92 (72)	NA	NA	NA
After school									
Intervention (ITT)	58/96 (60)	53/83 (64)	3.5	1.21 (0.39 to 3.72)	.74	58/86 (67)	6.7	1.60 (0.51 to 4.97)	.42
Intervention (PP)	25/48 (52)	31/48 (65)	12.6	2.68 (0.59 to 12.16)	.20	34/48 (71)	18.4	5.63 (1.16 to 27.22)	.03
Control	61/94 (65)	59/91 (65)	NA	NA	NA	60/92 (65)	NA	NA	NA
**Physical activity guidelines**									
Before school									
Intervention (ITT)	68/101 (67)	66/91 (73)	5.5	1.73 (0.50 to 6.00	.39	71/88 (81)	−0.6	0.77 (0.19 to 3.13)	.71
Intervention (PP)	33/48 (69)	36/48 (75)	6.6	1.87 (0.42 to 8.41))	.41	40/48 (83)	0.6	0.98 (0.18 to 5.23)	.98
Control	68/96 (71)	67/95 (71)	NA	NA	NA	78/92 (85)	NA	NA	NA
After school									
Intervention (ITT)	77/101 (76)	72/91 (79)	6.2	1.88 (0.54 to 6.53)	.32	79/88 (90)	10.8	2.47 (0.59 to 10.32)	.21
Intervention (PP)	37/48 (77)	39/48 (81)	7.4	1.99 (0.46 to 8.66)	.36	45/48 (94)	13.9	4.24 (0.68 to 26.48)	.12
Control	85/96 (89)	81/95 (85)	NA	NA	NA	84/92 (91)	NA	NA	NA

Abbreviations: ARD, absolute risk difference; ITT, intention to treat; NA, not applicable; OR, odds ratio; OSHC, Outside School Hours Care; PP, per protocol.

Individual guideline components showed the per-protocol effect was mainly driven by after-school screen time adherence; accredited services in the intervention group were more likely than services in the control group to meet this guideline at 12 months (OR, 5.63; 95% CI, 1.16-27.22; *P* = .03) (Table 2), with no significant between-group differences for physical activity guidelines.

### OSHC Staff Physical Activity–Related Behaviors

Staff at services in the intervention group demonstrated significantly greater use of physical activity–enabling behaviors during before-school care (Table 3). In ITT analyses, services in this group showed higher odds of positive staff behaviors during before-school care than those in the control group at 12 months (OR, 1.72; 95% CI, 1.27-2.33; *P* < .001). In per-protocol analyses, accredited services demonstrated significantly greater increases than services in the control group at both 3 months (OR, 1.55; 95% CI, 1.06-2.25; *P* = .02) and 12 months (OR, 3.00; 95% CI, 2.10-4.28; *P* < .001). No significant differences were observed for staff behaviors during after-school care.

**Table 3.** OSHC Staff Physical Activity–Related Behaviors at Baseline, 3 Months, and 12 Months

Behavior	Staff observations, median (IQR), %		ARD, %^a^	Group-by-time interaction baseline to 3 mo		Staff observations, median (IQR), %	ARD, %^a^	Group-by-time interaction baseline to 12 mo	
	Baseline	3 mo		OR (95% CI)	*P* value	12 mo		OR (95% CI)	*P* value
**Positive physical activity behaviors**									
Before school									
Intervention (ITT)	3.0 (0 to 14.3)	0 (0 to 13.7)	0.6	1.02 (0.75 to 1.40)	.88	7.0 (0 to 18.1)	4.4	1.72 (1.27 to 2.33)	<.001
Intervention (PP)	0 (0 to 10.5)	1.6 (0 to 14.6)	3.1	1.55 (1.06 to 2.25)	.02	10.0 (0 to 23.9)	9.3	3.00 (2.10 to 4.28)	<.001
Control	2.4 (0 to 14.1)	3.9 (0 to 10.3)	NA	NA	NA	0 (0 to 11.5)	NA	NA	NA
After school									
Intervention (ITT)	7.6 (2.5 to 16.3)	8 (2.6 to 19.3)	−0.8	0.93 (0.77 to 1.12)	.45	7.9 (2.5 to 18.0)	−0.1	0.96 (0.79 to 1.17)	.69
Intervention (PP)	12.3 (2.6 to 20.4)	13.0 (4.8 to 25.3)	0.9	1.08 (0.86 to 1.34)	.51	11.5 (4.3 to 20.9)	0.4	1.03 (0.82 to 1.30)	.78
Control	8.1 (0 to 17.2)	8.6 (2.1 to 20.2)	NA	NA	NA	8.4 (2.4 to 16.0)	NA	NA	NA
**Negative physical activity behaviors**									
Before school									
Intervention (ITT)	0 (0 to 0)	0 (0 to 0)	−0.2	0.87 (0.19 to 3.95)	.86	0 (0 to 0)	0.2	2.04 (0.32 to 12.91)	.45
Intervention (PP)	0 (0 to 0)	0 (0 to 0)	−0.1	1.47 (0.24 to 9.17)	.68	0 (0 to 0)	0.2	2.77 (0.32 to 24.23)	.36
Control	0 (0 to 0)	0 (0 to 0)	NA	NA	NA	0 (0 to 0)	NA	NA	NA
After school									
Intervention (ITT)	0 (0 to 0)	0 (0 to 0)	−0.1	0.84 (0.37 to 1.92)	.68	0 (0 to 0)	0	1.02 (0.44 to 2.39)	.96
Intervention (PP)	0 (0 to 0)	0 (0 to 0)	−0.2	0.7 (0.25 to 1.97)	.50	0 (0 to 0)	−0.1	0.85 (0.30 to 2.47)	.77
Control	0 (0 to 0)	0 (0 to 0)	NA	NA	NA	0 (0 to 0)	NA	NA	NA

Abbreviations: ARD, absolute risk difference; ITT, intention to treat; NA, not applicable; OR, odds ratio; OSHC, Outside School Hours Care; PP, per protocol.

^a^ ARDs were calculated as the change in aggregate staff observation percentage from baseline to follow-up in the intervention group minus the corresponding change in the control group. All other results calculated at the per-visit level.

Interrater reliability for SOSPAN observations was high. The median Cohen κ value was 0.92 (range, 0.42-1.00) with 47 of 48 variables above the predefined acceptability criterion (κ ≥0.60). Median percentage agreement was 97.8% (range, 88.%-100%) with 48 of 48 variables above the predefined acceptability criterion (≥80%).

### Observed Physical Activity and Screen Time Behaviors of Children During OSHC

In ITT analyses, children’s MVPA and recreational screen time did not differ significantly between groups during before- or after-school care at either time point (Table 4). In per-protocol analyses, children attending accredited services in the intervention group had higher MVPA than those attending services in the control group during before-school care at 12 months (IRR, 1.23; 95% CI, 1.05-1.43; *P* = .01). No significant differences were observed at 3 months for after-school MVPA or recreational screen time.

**Table 4.** Observed Physical Activity and Screen Time by Children During OSHC at Baseline, 3 Months, and 12 Months

Characteristic	No./total No. (%)		ARD, %	Group-by-time interaction baseline to 3 mo		No./total No. (%)	ARD, %	Group-by-time interaction baseline to 12 mo	
	Baseline	3 mo		IRR (95% CI)	*P* value	12 mo		IRR (95% CI)	*P* value
**Screen time**									
Before school									
Intervention (ITT)	1145/18 162 (6)	1032/15 796 (7)	0.6	0.95 (0.72 to 5.08)	.19	934/17 278 (5)	−0.6	0.97 (0.34 to 2.77)	.96
Intervention (PP)	723/9883 (7)	598/8325 (7)	0.3	1.11 (0.37 to 3.38)	.85	401/10 438 (4)	−3.1	0.29 (0.13 to 1.61)	.23
Control	1091/17 794 (6)	969/16 898 (6)	NA	NA	NA	1036/17 877 (6)	NA	NA	NA
After school									
Intervention (ITT)	3296/45 349 (7)	2200/41 461 (5)	−3.8	0.76 (0.33 to 1.72)	.51	2683/41 211 (6)	−0.8	1.31 (0.58 to 2.96)	.52
Intervention (PP)	1895/22 605 (8)	1153/21 871 (5)	−4.9	0.58 (0.23 to 1.47)	.25	1368/22 190 (6)	−2.3	0.89 (0.35 to 2.23)	.80
Control	2102/45 132 (5)	2801/43 155 (7)	NA	NA	NA	1930/40 644 (5)	NA	NA	NA
**Physical activity (MVPA)**									
Before school									
Intervention (ITT)	6534/18 162 (36)	5758/15 796 (36)	0.6	1.03 (0.90 to 1.17)	.69	6810/17 278 (39)	2.8	1.08 (0.95 to 1.23)	.24
Intervention (PP)	3532/9883 (36)	3210/8325 (39)	3.0	1.15 (0.98 to 1.34)	.08	4480/10 438 (43)	6.5	1.23 (1.05 to 1.43)	.01
Control	5995/17 794 (34)	5670/16 898 (34)	NA	NA	NA	6137/17 877 (34)	NA	NA	NA
After school									
Intervention (ITT)	19 149/45 349 (42)	18 128/41 461 (44)	3.5	1.10 (1.00 to 1.21)	.06	18 270/41 211 (44)	1.1	1.04 (0.94 to 1.14)	.46
Intervention (PP)	9681/22 605 (43)	9426/21 871 (43)	2.3	1.10 (0.98 to 1.24)	.12	10 312/22 190 (47)	2.6	1.08 (0.96 to 1.22)	.21
Control	18 969/45 132 (42)	17 247/43 155 (40)	NA	NA	NA	17 493/40 644 (43)	NA	NA	NA

Abbreviations: ARD, absolute risk difference; IRR, incidence rate ratio; ITT, intention to treat; MVPA, moderate-to-vigorous physical activity; NA, not applicable; OSHC, Outside School Hours Care; PP, per protocol.

Interrater reliability for SOPLAY observations was high. The median intraclass correlation coefficient (ICC) was 0.90 (range, 0.76-0.93), with 9 of 10 variables meeting the predefined acceptability criterion (ICC ≥ 0.80).

### Screen and Physical Activity Equipment Availability

In ITT analyses, services in the intervention group showed higher odds of screen availability than those in the control group during before-school care at 3 months (OR, 1.34; 95% CI, 1.05-1.71; *P* = .02; eTable 2 in Supplement 2). In per-protocol analyses, services in the intervention group demonstrated lower odds of screen availability than services in the control group during after-school care at both 3 months (OR, 0.75; 95% CI, 0.62-0.90; *P* = .002) and 12 months (OR, 0.82; 95% CI, 0.68-0.99; *P* = .04).

For physical activity equipment, services in the intervention group showed lower odds of availability than those in the control group during before-school care at 3 months (ITT OR, 0.65; 95% CI, 0.54-0.79; *P* < .001; per-protocol OR, 0.73; 95% CI, 0.58-0.91; *P* = .006) but higher odds of availability during before-school care at 12 months (ITT OR, 1.21; 95% CI, 1.00-1.46; *P* = .048; per-protocol OR, 1.27; 95% CI, 1.03-1.58; *P* = .03), as well as during after-school care at 12 months in both ITT (OR, 1.16; 95% CI, 1.04-1.30; *P* = .006) and per-protocol analyses (OR, 1.24; 95% CI, 1.10-1.41; *P* = .001; eTable 2 in Supplement 2).

## Discussion

To our knowledge, this is the largest trial of a physical activity and screen time intervention in extended education settings globally, involving 197 OSHC services caring for approximately 10 000 children. In ITT analyses, there were no significant effects on guideline adherence or children’s observed physical activity or recreational screen time. However, services in the intervention group showed greater before-school staff physical activity–promoting behaviors and higher physical activity equipment availability at 12 months. Although 80% of intervention services registered, only 48% achieved accreditation. In per-protocol analyses, accredited services were more likely to meet guideline components at 12 months, with improvements in staff behaviors, children’s before-school MVPA, screen availability, and physical activity equipment availability.

The contrasting ITT and per-protocol findings indicate that implementation reach was central to impact. Services that completed the program showed consistent changes across policy alignment, screen availability, equipment availability, staff behaviors, and children’s physical activity, whereas incomplete implementation diluted population-level effects. This pattern is consistent with the intervention’s voluntary, system-level design, drawing on the Theoretical Domains Framework^20^ to target barriers and enablers identified through consultation with the OSHC sector, including staff knowledge, skills, confidence, routine decision-making, professional role identity, environmental context, and resources. Before school, where baseline activity promotion was lower, effects were strongest for staff behaviors and were accompanied by a 23% increase in children’s MVPA. After school, where baseline staff engagement was higher, changes were primarily environmental, including reduced screen availability, increased physical activity equipment availability, and improved screen time adherence. These patterns align with previous evidence identifying educator behaviors, environmental cues, physical activity equipment availability, and scheduling practices as key levers for movement-supportive practice in school-aged care settings.^14,25^

Child-level behavior changes were more modest than service-level changes, particularly after school. This is unsurprising given that the intervention targeted service-level factors rather than individual child behaviors. In OSHC, multiple activities are offered simultaneously and children choose which to participate in.^26,27^ When a service increases physical activity opportunities or reduces screen availability, this changes what is on offer but does not require every child to be active or avoid screens. The national guidelines and Activated OSHC program appropriately focus on what services can control (ensuring active play is scheduled and limiting screen time) rather than mandating individual children’s choices. In addition, with child behaviors analyzed at the service level, statistical power for detecting small but potentially meaningful individual changes was limited despite observing several thousand children.

The present findings are broadly consistent with accreditation-based health behavior change interventions in analogous organizational settings. In community sports, a multicomponent accreditation program combining policy development, staff training, printed resources, audit feedback, and project officer support produced a significant reduction in risky alcohol consumption among club members in regional Australian football clubs compared with controls.^28^ Sustained implementation of alcohol management practices following a similar accreditation model has also been demonstrated.^29,30^ In school settings, accreditation-linked policy approaches to sun protection have similarly demonstrated improvements in staff role-modeling behaviors and children’s sun-safe practices.^31,32,33^ A common feature across these programs is that meaningful behavior change was most evident among organizations that progressed to higher levels of accreditation, a pattern consistent with the per-protocol findings of the present trial, where effects on service-level guideline adherence, staff behavior, and children’s physical activity were concentrated in services that achieved Activated OSHC accreditation.

### Implications and Future Directions

These findings have immediate practice and policy implications. The Activated OSHC program provides a structured, feasible pathway for services to align with national guidelines using a relatively low-resource model suitable for scale-up. However, variable uptake underscores that making programs available is insufficient; implementation support is essential.

Future efforts should test strategies to enhance uptake, such as implementation facilitation, financial incentives, peer learning networks, or integration into existing funding and quality assurance systems, as well as the association of these strategies with the cost of delivery. Research should examine equity of uptake across service types, sustainability of accreditation effects, and whether enhanced implementation supports improve population-level impact. Given this setting’s international reach and the transferability of the accreditation model, replication trials in other countries would be valuable.

This trial demonstrates that achieving population health impact through voluntary, systems-level interventions requires addressing both intervention effectiveness and implementation reach. The Activated OSHC program succeeded in changing practice where implemented; the next challenge is optimizing implementation.

### Strengths and Limitations

Key strengths include the trial’s scale for this setting, multistate reach, rigorous methodology with blinded outcome assessment and validated direct observation tools, high retention (92% at 12 months), and pragmatic design testing the intervention under usual conditions. The codesigned intervention and long-standing sector partnership enhanced ecological validity.

Limitations include the geographic recruitment imbalance between study sites. The absence of individual-level child data, while enhancing ecological validity and sample representativeness,^34^ limited statistical power for detecting child-level change and precluded examination of individual trajectories. Potential reactivity to data collection was minimized through unannounced visits but cannot be eliminated. In addition, behavior observed at 1 before-school and 1 after-school session at each time point may not fully represent typical behavior. The screen time equipment item was added to the SOPLAY protocol specifically for this study; as this item has not undergone independent psychometric validation, its measurement properties remain uncertain, although identical administration across intervention and control conditions means any error is unlikely to have differentially affected outcomes. While 48% of intervention services achieved accreditation within 12 months, 52% of intervention services did not, limiting population-level impact. Per-protocol analyses may have introduced selection bias and postrandomization confounding and have reduced generalizability.

## Conclusions

This RCT provides evidence that accreditation can drive meaningful improvements in movement-supportive practices in extended education settings at scale. Among engaged services, the intervention is suggestive of benefits across staff behaviors, environmental conditions, guideline adherence, and children’s physical activity through a scalable, low-resource model. Although variable uptake limited population-level effects, the findings point to accreditation as a promising mechanism for system-level change among the tens of millions of children attending organized care globally. The results provide timely, policy-relevant evidence for national efforts to improve children’s physical activity and screen time behaviors, with useful suggestions for implementation strategies to maximize reach and impact at scale. Government actions to do so provide further opportunities for health and education systems to contribute to the evidence base, learn, and improve the impact of health promotion initiatives in this setting.^35^

## Notes

Supplement 1. Trial Protocol and Statistical Analysis Plan

Supplement 2. **eTable 1.** Comparison Between Accredited and Unaccredited Intervention Services Baseline Characteristics **eTable 2.** Screen and Physical Activity Equipment Availability During Sessions at Baseline, 3 Months and 12 Months **eFigure 1.** Activated OSHC Training Modules **eFigure 2.** Activated OSHC Resources Available to OSHC Services **eFigure 3.** Out of School Hours Care Services Adherence to OSHC Sector Physical Activity and Screen Time Guidelines at Baseline, 3 Months and 12 Months

Supplement 3.
Data Sharing Statement
