# Supplementary material for: A Policy-Based Intervention for Physical Activity and Screen Time in Childcare Outside School Hours: A Cluster Randomized Clinical Trial
Source: JAMA Netw Open. 2026 Oct 5;9(10):e2636741. doi: 10.1001/jamanetworkopen.2026.36741 (PMC13639758; doi:10.1001/jamanetworkopen.2026.36741)
Supplement: Supplementary file 1 [file jamanetwopen-e2636741-s001.pdf]

Clinical Trial Protocol

Administrative information

Title:
A multisite guideline implementation randomised controlled trial to improve physical activity and screen time in Out of School Hours Care

Trial registration:
This trial will be registered in the Australian New Zealand Clinical Trials Registry (ANZCTR)

Protocol version:
1 (29/03/2022)

Trial Sponsor:
University of South Australia

Coordinating Investigator:
Prof Carol Maher, PhD.

Other Investigators:
Prof Adrian Esterman, PhD. University of South Australia
Dr Rachel Curtis, PhD. University of South Australia
Dr Rosa Virgara, PhD. University of South Australia
Assoc Prof Hayley Christian, PhD. University of Western Australia/Telethon Kids Institute
Prof Luke Wolfenden, PhD. The University of Newcastle/Hunter New England Population Health
Dr Nicole Nathan, PhD. The University of Newcastle/Hunter New England Population Health
Assoc Prof Svetlana Bogomolova, PhD. Flinders University
Assoc Prof Lucy Lewis, PhD. Flinders University
Dr Rachel Milte, PhD. Flinders University
Dr Rebecca Stanley, PhD. The University of Wollongong
Prof Anthony Okely, PhD. The University of Wollongong
Dr Dylan Cliff, PhD. The University of Wollongong
Prof Richard Rosenkranz, PhD. Kansas State University
Mandy Richardson. Out of School Hours Care SA Association
Kylie Brannelly. National Outside School Hours Services Alliance

Research ethics approval:
Ethics approval will be sought from the Human Research Ethics Committee of the University of South Australia (trial sponsor and grant administrator).

The trial will be conducted at two additional sites: University of Western Australia and University of Newcastle. Therefore, ethics approval will be sought from the University of Western Australia and University of Newcastle Human Research Ethics Committees, based on the approval first granted by the UniSA Human Research Ethics Committee. In addition, some participants will be from Out of School Hours Care (OSHC) services run by schools. The project will obtain approval by the Department of Education and Catholic Education/Catholic Schools in SA, WA, and NSW.

The trial will be conducted in compliance with the National Statement on Ethical Conduct in Human Research and conditions of the Human Research Ethics Committees

Introduction

Rationale:

Children's physical activity and screen time behaviours have wide-ranging impacts on their physical health and wellbeing [1]. The prevalence of Australian childhood overweight and obesity has quadrupled since the 1960s [2], and 1 in 5 children have emotional and mental health concerns [3]. Less than half of Australian children meet the physical activity guideline of 60 minutes per day and only one third meet the screen time guideline of <2h per day [4]. New approaches to improving Australian children's physical activity and screen time patterns are needed. Nearly half a million 5–12-year-old Australian school children attend OSHC every week. With before-school care sessions lasting around 2 hours, after-school care 3.5 hours and vacation care up to 11 hours per day, the activities that children undertake at OSHC play a crucial role in shaping whether they meet daily physical activity and screen time recommendations.

Currently, physical activity and screen time practices in OSHC are highly variable and lack guidance from clear policy [5]. An in-person audit of randomly selected SA and NSW OSHC services [5] and national survey of n=566 OSHC directors (manuscript under review) found that daily recreational screen time was as high as 5.5 hours per day in some OSHC services, and some OSHC services offered no daily physical activity. OSHC directors' awareness of the Australian 24h physical activity and screen time recommendations was low (only 16%). Additionally, there were widely contrasting views amongst OSHC directors regarding the role of OSHC in helping children meet 24h physical activity and screen time recommendations.

Throughout 2019-2020, we led an extensive national stakeholder consultation process to co-design evidence based Australian OSHC physical activity and screen time guidelines. The guidelines are expressed in terms of scheduled time for physical activity using a 3:1 ratio (Figure 1) and are intended to support children to achieve 15 minutes of physical activity before school, 30 minutes after school and 1 hour in vacation care [6]. Our 2020 survey of Australian OSHC directors indicated strong support for the guidelines but only 41% of participating services met the guidelines.

This randomised controlled trial (RCT) will provide the "Activated OSHC" online training and accreditation program, aimed at improving adherence to the new guidelines. This project has substantial capacity to improve children's daily activities and health. The RCT will be conducted in 192 OSHC services, reaching thousands of children, and will inform dissemination of the guidelines to thousands of OSHC services, potentially benefiting hundreds of thousands of Australian children. In addition, ACECQA (Australian Children's Education & Care Quality Authority – the federal agency overseeing the accreditation of OSHC services throughout Australia), has stated that the guidelines have strong potential to be embedded into their National Quality Framework in the future.

Session	Physical Activity	Recreational Screen Time
**Before School Care**	Schedule **45 minutes** of time for children to engage in a variety of physical activities including energetic play. **More is better**.	Discourage children from engaging with screens, with total screen time no more than **30 minutes. Less is better**.
**After School Care**	Schedule **90 minutes (1.5 hrs)** of time for children to engage in a variety of physical activities including energetic play. **More is better**.	Discourage children from engaging with screens, with total screen time no more than **60 minutes. Less is better**.
**Vacation Care**	Throughout the day, schedule **at least 3 hours** of time for children to engage in a variety of physical activities including energetic play. **More is better**.	Discourage children from engaging with screens, with total screen time no more than **2 hours** over the entire day. **Less is better**.

Figure 1. Australian OSHC sector physical activity and screen time guidelines.

Aims:
This study aims to examine the effectiveness, cost-effectiveness, acceptability, and feasibility of implementing the Activated OSHC program –an online training and accreditation program aimed at increasing implementation of the national OSHC physical activity and screen time guidelines.

Research questions:
This study has five research questions:

1. What is the effectiveness of the Activated OSHC program for increasing the proportion of time children spend being physically active at OSHC?
2. What is the effectiveness of the Activated OSHC program for reducing the proportion of time children spend in recreational screen time at OSHC?
3. What is the effectiveness of the Activated OSHC program for increasing the proportion of OSHC services meeting the new evidence based Australian OSHC sector physical activity and screen time guidelines?
4. What is the cost-effectiveness of the Activated OSHC program?
5. What is the acceptability and feasibility of the Activated OSHC program?

Methods: Design, participants, allocation, and intervention

Design:

This is a 12-month randomised controlled trial of the Activated OSHC program that aims to assist OSHC services to develop and implement a physical activity and screen time policy. The unit of randomisation and unit of analysis are at the OSHC service level. Figure 2 shows the CONSORT diagram. 162 metropolitan and 30 regional OSHC services across South Australia, Western Australia and New South Wales will be randomly allocated to the intervention or control group. The control group will receive the intervention at the conclusion of the study. OSHC directors will complete online survey assessments at baseline, 3 and 12 months. Children attending OSHC will be observed at baseline, 3 and 12 months. A subsample of directors and OSHC staff members from the intervention group will complete an online interview at 12 months. Recruitment will begin in June 2022.

Participants:
192 OSHC services (162 metropolitan and 30 regional) will be recruited. Eligible OSHC services will be identified by downloading a list of care services in SA, NSW, and WA from the Australian Children's Educational Care Quality Authority (ACECQA) website. Eligible OSHC services must provide both before school care and after school care. Additionally, they must be located within 120 km of Adelaide, Perth, or Newcastle, or be located in one of the following regional areas: SA: Riverland, Eyre and Western, Far North, Yorke and Mid-North; NSW: Central Coast and Hunter region; WA: Augusta-Margaret River, Busselton, Bunbury. The OSHC director must speak English and have internet access.

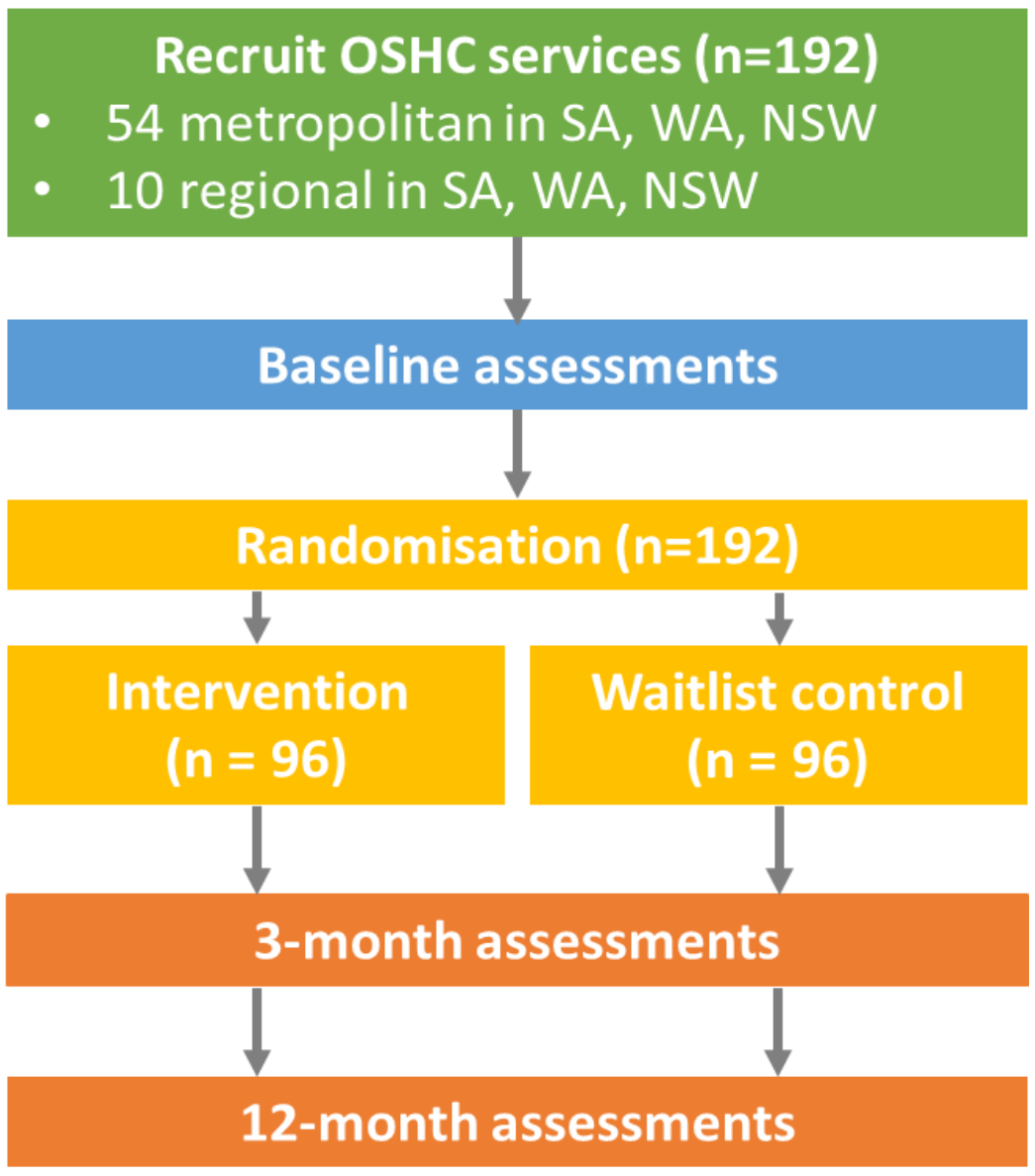

Figure 2. CONSORT flow chart.

A rolling recruitment and enrolment process will take place. Metropolitan services will be divided into socioeconomic status tertiles. 20 randomly selected services per SES tertile within 50kms of each study site will initially be invited via email, followed by a

phone call after a week to ensure the invitation was received. Up to three calls will be made to determine interest in participating. Eligibility of services will be confirmed during telephone contact. Additional services will be invited each month, increasing the sampling range to 75km, 100km, and 120km as required until 54 in each state (total 162) are recruited. For practical reasons, convenience sampling will be applied in regional areas. Regional sites will be invited within geographic clusters (so that data collection is feasible) until 10 services in each state (total n=30) are recruited. NSW sites will initially be recruited within the Hunter New England Local Health District with additional sites recruited outside this area if required.

Among eligible OSHC services, all directors who speak English and have internet access will be eligible to participate in the online surveys. All children at participating OSHC services will be eligible to be observed. Directors (n=15) and staff (n=15) from services allocated to the intervention group, who speak English and have access to an internet enable device with microphone/webcam will be eligible to participate in the interviews.

Consent:
All participants will provide consent prior to enrolment. An invitation email will be sent to OSHC directors to invite them and their OSHC service to participate. This will include the information sheet and a link to register their interest via an online REDCap form. Interested participants will receive a phone call to confirm eligibility and eligible participants will receive an email link to complete an online consent form. For participating OSHC services, an information letter will be provided to parents of children attending the program, which will explain the study and contain an opt out slip. The opt out slip will be returned to the OSHC service if they wish to exclude their child from observation. Additionally, OSHC directors and educators will be sent an invitation email or letter to participate in the interviews, which will include an information sheet and consent form, which will be returned via email.

OSHC services/directors/staff can withdraw from the study at any time by emailing or telephoning the study team. Their withdrawal will be acknowledged. Following this, they will not be contacted again by the research team and no further information will be collected. Data collected to date will be used for analysis unless participants request to withdraw their data via email or telephone. Withdrawn participants will not receive the honoraria outlined in the information sheets.

Children/parents may withdraw consent to be observed at any time by emailing or telephoning the study team. Their withdrawal will be acknowledged. Following this, they will not be contacted by the research team and children will not be included in future observations. Any data already collected will be retained as it will not be possible to identify and exclude that data.

Dates and reasons for withdrawal (if provided) will be recorded. Withdrawn participants will not be replaced.

Allocation and blinding:
Following baseline data collection, REDCap will be used to randomise OSHC services in a 1:1 allocation ratio to either the intervention or control group. Randomisation will be stratified based on state and SES tertile. Block randomisation will be used to ensure group allocation is equal.

Participants cannot be blinded to condition. Data collection will be conducted by research staff blinded to group allocation.

Honoraria:
Participating OSHC services will receive a $250 HART Sport voucher to purchase equipment for the OSHC service or may opt for an equivalent payment for an active OSHC excursion or relevant staff

professional development, by negotiation. OSHC directors and staff who participate in the interviews will receive a $50 Amazon voucher.

Intervention:

The intervention components have been developed using the Theoretical Domains Framework (TDF) – an integrative implementation framework that synthesises behaviour change constructs that enable or pose barriers to the implementation of evidence-based practice [7].This framework has been widely used in effective clinical practice change interventions and has been previously applied by the research team to design implementation strategies to improve adherence to sector physical activity policy in schools [8,9]. The implementation strategies are directly linked to enablers and barriers identified through extensive consultation with the OSHC sector and mapped to TDF constructs. Participants will be provided with the following resources to assist in implementing the physical activity and screen time guidelines:

a) **"Becoming an Activated OSHC" manual**: This manual provides education on the new physical activity and screen time guidelines, recommendations and examples of how to schedule physical activity and screen time, and practical tips on how to encourage active play. It also describes the process of completing the intervention to become an "Activated OSHC" (i.e., all staff to complete training modules, develop an approved physical activity and screen time policy).

b) **Online training resources**: A series of four online training modules will be provided –
    1. *Importance of Change* describes the benefits of becoming an "Activated OSHC". It describes the physical activity and screen time guidelines, the benefits of physical activity for children, and the importance of staff role-modelling of healthy physical activity behaviours. It identifies and addresses barriers and enablers that OSHC educators face when trying to get children to engage in more physical activity.
    2. *Making physical activity fun* describes how to make physical activity fun and increase physical activity scheduling, including practical tips on increasing children's participation (e.g., avoiding lines or elimination games) and enabling children to choose how to be active (e.g., which games and how they're played).
    3. *Savouring screen time* describes how to reduce screen time (e.g., when to schedule screen time) and how to provide active screen-based games rather than passive screens. It also describes how to inform and involve families in the new scheduling changes.
    4. *Becoming an Activated OSHC* examines staff knowledge of the OSHC physical activity and screen time guidelines, including scheduling, guideline limits, and ways to increase physical activity and reduce screen time.

c) **Posters**: Posters reminding staff, children and families about the guidelines will be displayed in OSHC services. These resources will help address key barriers and enablers identified in the national survey (e.g., potential resistance from the children to changing screen time practices).

d) **Family communication resources**: OSHC services will received resources (brochures plus newsletter-suited text) they can use to help families understand OSHC programming changes, and support integration into positive physical activity and screen time practices at home.

The intervention group will receive the intervention after completing the baseline assessments. The control group will receive the intervention at the conclusion of the study. No restrictions regarding concomitant training and resources will be applied to either group.

Methods: Outcomes

Outcomes will be collected at baseline (prior to randomisation) and at 3- and 12-months post-baseline. Directors will complete an online survey at all timepoints. Children will be observed at all timepoints. Directors and staff from the intervention group will complete qualitative interviews after 12 months.

Primary Outcomes:

Children's physical activity and recreational screen time behaviour will be measured using direct observation, the gold standard in settings-based physical activity measurement [10]. The SOPLAY direct observation tool (System for Observing Play and Leisure Activity in Youth) is a reliable and validated method [11,12], in which two assessors conduct continuous visual scans of children's play zones for an entire care session. Every 15 minutes for the duration of the care session, they describe the conditions of each play zone (e.g., whether it has physical activity equipment present) and the number of children engaging in sedentary activities, walking or vigorous activities. The directly observed data will be used to calculate the proportion of OSHC sessions spent in physical activity and recreational screen time. Children who are not participating will be asked to wear a clearly visible coloured lanyard during the observation period, and they will not be included in the observation.

To avoid observation bias, OSHC services will be advised of a 2-week window in which each assessment will occur, but the assessment day will be unannounced. Before and after school assessments will be completed on different days. Follow-up assessments will be conducted on the same day of the week as the baseline assessment where possible. To ensure the safety of research assistants travelling for site visits and working outdoors, assessments will not occur on catastrophic fire danger days, in extreme heat (>40 degrees) or in extreme storms (hail, lightning, wind). COVID safe measures will be adhered to, as outlined in the information sheet and COVID19 safety plan.

Secondary outcomes:

Physical activity and recreational screen time programming will be collected using an OSHC director-reported instrument we have previously developed and validated (r=0.40 for physical activity, and r=0.73 for screen time, relative to direct observation) [5]. These data will be used to determine whether services' programming meets the guidelines (i.e., scheduled time for before-school physical activity ≥45mins; before-school screen time ≤30mins; after-school physical activity ≥90mins; after-school screen time ≤60mins).

Educators' support of children's physical activity will be collected using the System for Observing Staff Promotion of Activity and Nutrition (SOSPAN) direct observation tool [13]. Alongside each SOPLAY visual scan, a scan is performed categorising the presence and behaviour of staff in relation to physical activity (engaged, instructing, promoting, discouraging, withholding, disabling behaviours, or off-task). The SOSPAN has strong to excellent validity and inter-observer reliability [31]. Data will be used to determine the proportion of care sessions that OSHC educators demonstrate physical activity supporting behaviours.

OSHC service characteristics will include

- Years in operation
- The average number of children attending per day
- Socioeconomic status ('Index of Community Socio-Educational Advantage' rating)

- ACECQA Meeting NQS, NQS ratings
- Service governance
- Budget for sports/active play equipment
- Spending on sports/active play equipment
- Use of personal electronic devices in OSHC
- Whether the service currently has a written physical activity or screen time policy
- Whether the service mentions physical activity in marketing or promotion information (e.g., pre-enrolment information pack, welcome pack, advertising)
- At baseline, OSHC directors will provide copies of any current physical activity and screen time policies and any marketing material they may use to describe their OSHC as promoting physical activity

OSHC staff characteristics will include

- Director age, gender, qualifications, and years of service
- Number of staff
- Staff age, gender, and qualifications
- Staff turnover in the past 12 months
- Staff recruitment in the past 12 months
- Physical-activity-related professional development training in last 12 months

Implementation process outcomes:

Resource uptake: Logs of how the online resources were used will be downloaded at 12 months.

Determinants of implementation behaviour will be measured using purpose-designed items (mapped onto the TDF framework) that assess various domains relating to implementation of physical activity and screen time in OSHC (knowledge, skills, role and identity, beliefs about capabilities, beliefs about consequences, intentions, environmental context and resources, social influences, behavioural regulation).

Acceptability, appropriateness, and feasibility: OSHC directors in the intervention group only (n=96) will evaluate specific components of the program, for example which components they used, how important the components were, and how satisfied they were with components. They will also complete a discrete choice experiment, where they will be asked to indicate their preferences for a series of hypothetical packages of interventions to improve adherence to the guidelines. OSHC directors will be asked to make choices between a series of pairwise packages of interventions providing different levels across the included attributes.

Qualitative semi-structured interviews with a subsample of OSHC directors (n=15) and staff (n=15) in the intervention group will explore participants' perceptions and experiences of the intervention. Interviews will be conducted and recorded via Zoom and verbatim transcripts will be generated and thematically coded.

Methods: Data analysis and management

Statistical methods:

Analyses will be performed following intention to treat principles, with the OSHC service as the unit of analysis. If assumptions warrant it, multiple imputation will be used to replace missing data. Analyses plan for the 5 identified research questions:

1. Mixed-effects Poisson regression will be used to determine group x time changes in children's physical activity. The models will be adjusted by the stratification factors (state and SES tertile).
2. Mixed-effects Poisson regression will be used to determine group x time changes in children's screen time behaviours.
3. Mixed-effects multivariable logistic regression will be used to determine group x time changes in the proportion of intervention and control services with programming schedules that meet the OSHC sector physical activity and screen time guidelines.
4. Cost effectiveness will be analysed using the incremental cost-effectiveness ratio, based on improvements in the primary outcomes. An assessment of the sensitivity of the results obtained with respect to variation in measured resource use, effectiveness and/or unit costs will be undertaken using appropriate one-way and multi-way sensitivity analyses.
5. Quantitative acceptability/feasibility data will be analysed descriptively; qualitative data will be transcribed and analyses thematically.

Data management:

Data will be owned by the University of South Australia. Data will be re-identifiable. Master codes to reidentify participants will be stored in the REDCap study database. This will be accessible only by members of the project team who will require specific authorization, as well as a username and password to access REDCap. Data on REDCap is stored locally on UniSA servers.

Data will be collected and stored on REDCap. Data from paper forms used during direct observation will be entered into REDCap. During the study, paper forms will be stored in locked filing cabinets in SA (Chief Investigator Maher's office C741 Centenary building, University of South Australia), WA (Level 6, East wing of the Telethon Kids Institute, Perth Children's Hospital) and NSW (Data storage room, Level 1 Booth Building, Hunter New England Population Health). At the conclusion of the study, paper copies will be sent to the University of South Australia for storage. Zoom recordings and transcripts will be stored on the UniSA server. All data will be additionally stored on the UniSA server, in a password-protected folder (outside of the REDCap interface) at the conclusion of the study.

Data will be stored for 15 years after the completion of the project, in line with Australian Code for the Responsible Conduct of Research and the University of South Australia's human research guidelines for clinical trials. Data will be destroyed after the data retention period has elapsed upon obtaining permission from the Dean of Research at the University of South Australia. Paper copies will be destroyed using confidential bin disposal. Computer files will be erased.

References

1. Reiner M, Niermann C, Jekauc D, et al. Long-term health benefits of physical activity – A systematic review of longitudinal studies. *BMC Public Health.* 2013, 13(1):813.
2. Ho NS, Olds T, Schranz N, et al. Secular trends in the prevalence of childhood overweight and obesity across Australian states: A meta-analysis. *Journal of Science and Medicine in Sport.* 2017, 20(5):480-488.
3. Bayer JK, Ukoumunne OC, Lucas N, et al. Risk factors for childhood mental health symptoms: National longitudinal study of Australian children. *Pediatrics.* 2011, 128(4):e865-879.
4. Schranz N, Glennon V, Evans J, et al. Results from Australia's 2018 report card on physical activity for children and youth. *Journal of Physical Activity and Health.* 2018, 15(S2):S315-S317.
5. Maher C, Virgara R, Okely T, et al. Physical activity and screen time in out of school hours care: an observational study. *BMC Pediatrics.* 2019, 19(1):283.

6. Virgara R, Lewis L, Phillips A, et al. Development of Australian physical activity and screen time guidelines for Outside Schofol Hours Care: an international Delphi study. *International Journal of Behavioral Nutrition and Physical Activity.* 2021;18:3.
7. Michie S, Johnston M, Abraham C, et al. Making psychological theory useful for implementing evidence based practice: A consensus approach. *Quality and Safety in Health Care.* 2005;14(1):26-33.
8. Nathan N, Wiggers J, Bauman AE, et al. A cluster randomised controlled trial of an intervention to increase the implementation of school physical activity policies and guidelines: Study protocol for the physically active children in education (PACE) study. *BMC Public Health.* 2019;170.
9. Sutherland RL, Nathan NK, Lubans DR, et al. An RCT to facilitate implementation of school practices known to increase physical activity. *American Journal of Preventive Medicine.* 2017;53(6):818-828.
10. Sirard JR, Pate RR. Physical activity assessment in children and adolescents. *Sports Medicine*. 2001;31(6):439-54.
11. McKenzie TL, Marshall SJ, Sallis JF, et al. Leisure-time physical activity in school environments: An observational study using SOPLAY. *Preventive Medicine*. 2000;30(1):70-77
12. Saint-Maurice PF, Welk G, Ihmels MA, et al. Validation of the SOPLAY direct observation tool with an accelerometry-based physical activity monitor. *Journal of Physical Activity and Health*. 2011;8(8):1108-1116.
13. Weaver RG, Beets MW, Webster C, et al. System for Observing Staff Promotion of Activity and Nutrition (SOSPAN). *Journal of Physical Activity and Health*. 2014;11(1):173-185.
