# Supplementary material for: A Policy-Based Intervention for Physical Activity and Screen Time in Childcare Outside School Hours: A Cluster Randomized Clinical Trial
Source: JAMA Netw Open. 2026 Oct 5;9(10):e2636741. doi: 10.1001/jamanetworkopen.2026.36741 (PMC13639758; doi:10.1001/jamanetworkopen.2026.36741)
Supplement: Supplementary file 2 [file jamanetwopen-e2636741-s002.pdf]

Supplementary Online Content

Maher C, Wolfenden L, Nathan N, et al. A policy-based intervention for physical activity and screen time in Childcare Outside School Hours: a cluster randomized clinical trial. *JAMA Netw Open.* 2026;9(10):e2636741.
doi:10.1001/jamanetworkopen.2026.36741

eTable 1. Comparison Between Accredited and Unaccredited Intervention Services Baseline Characteristics

eTable 2. Screen and Physical Activity Equipment Availability During Sessions at Baseline, 3 Months and 12 Months

eFigure 1. Activated OSHC Training Modules

eFigure 2. Activated OSHC Resources Available to OSHC Services

eFigure 3. Out of School Hours Care Services Adherence to OSHC Sector Physical Activity and Screen Time Guidelines at Baseline, 3 Months and 12 Months

This supplementary material has been provided by the authors to give readers additional information about their work.

© 2026 Maher C et al. *JAMA Network Open.*

eTable 1. Comparison Between Accredited and Unaccredited Intervention Services Baseline Characteristics

	Intervention - accredited		Intervention - unaccredited		p value
**Services**	**n**	**(%)**	**n**	**(%)**	
All	48	(100)	53	(100)	0.096*
New South Wales	3	(6.2)	10	(18.9)	
South Australia	27	(56.2)	21	(39.6)	
Western Australia	18	(37.5)	22	(41.5)	
**Location**	**n**	**(%)**	**n**	**(%)**	
Metropolitan	47	(97.9)	43	(81.1)	0.007*
Regional	1	(2.1)	10	(18.9)	
**SES tertile, n (%)**	**n**	**(%)**	**n**	**(%)**	
High	17	(35.4)	12	(22.6)	0.366*
Mid	13	(27.1)	17	(32.1)	
Low	18	(37.5)	24	(45.3)	
**Service governance**	**n**	**(%)**	**n**	**(%)**	
Third-party provider	21	(43.8)	41	(77.4)	<0.001*
Run by school	27	(56.2)	12	(22.6)	
**Service size**	**median**	**[IQR]**	**median**	**[IQR]**	
Daily attendance	50	[30, 68]	35	[30, 50]	0.042^
Staff count	8	[6, 12]	5	[3, 10]	0.015^

Note: * denoted Chi-square test for categorical variables. ^ denoted Mann-Whitney U test for count variables.

© 2026 Maher C et al. *JAMA Network Open.*

eTable 2. Screen and Physical Activity Equipment Availability During Sessions at Baseline, 3 Months and 12 Months

	Baseline		3 months		ARD	Group x time interaction baseline to 3 months			12 months		ARD	Group x time interaction baseline to 12 months		
	%	n/N	%	n/N	%	OR	95% CI	p	%	n/N	%	OR	95% CI	p
**Screens available**														
Before school														
Intervention (ITT)	12.0	482/4015	13.6	440/3238	3.8	1.34	1.05, 1.71	0.018	11.3	340/3010	-0.3	1.11	0.86, 1.42	0.429
Intervention (PP)	12.4	251/2021	11.3	192/1702	1	0.93	0.70, 1.24	0.612	9.2	158/1726	-2.8	0.79	0.59, 1.06	0.112
Control	12.6	513/4075	10.4	358/3442					12.2	383/3150				
After school														
Intervention (ITT)	11.3	945/8384	10.5	784/7487	-0.8	0.90	0.77, 1.05	0.189	11.1	763/6849	0.5	1.13	0.96, 1.33	0.137
Intervention (PP)	12.8	523/4071	11.2	421/3772	-1.7	0.75	0.62, 0.90	0.002	10.5	386/3673	-1.7	0.82	0.68, 0.99	0.037
Control	10.6	855/8066	10.6	794/7457					10.0	658/6593				
**Physical activity equipment available**														
Before school														
Intervention (ITT)	18.1	726/4015	14.2	461/3238	-4.5	0.65	0.54, 0.79	<0.001	21.2	639/3010	2.3	1.21	1.00, 1.46	0.048
Intervention (PP)	17.5	353/2021	14.2	241/1702	-3.9	0.73	0.58, 0.91	0.006	23.3	402/1726	5	1.27	1.03, 1.58	0.028
Control	15.5	632/4075	16.1	555/3442					16.3	515/3150				
After school														
Intervention (ITT)	27.2	2278/8384	26.2	1958/7487	0.4	1.04	0.93, 1.15	0.519	29.8	2039/6849	3	1.16	1.04, 1.30	0.006
Intervention (PP)	27.6	1123/4071	27.8	1050/3772	1.6	1.09	0.96, 1.23	0.191	31.9	1172/3673	4.8	1.24	1.10, 1.41	0.001
Control	26.0	2099/8066	24.6	1837/7457					25.6	1687/6593				

Note: ARD = Absolute Risk Difference, OR = Odds Ratio, ITT = intention to treat, PP = per protocol

eFigure 1. Activated OSHC Training Modules

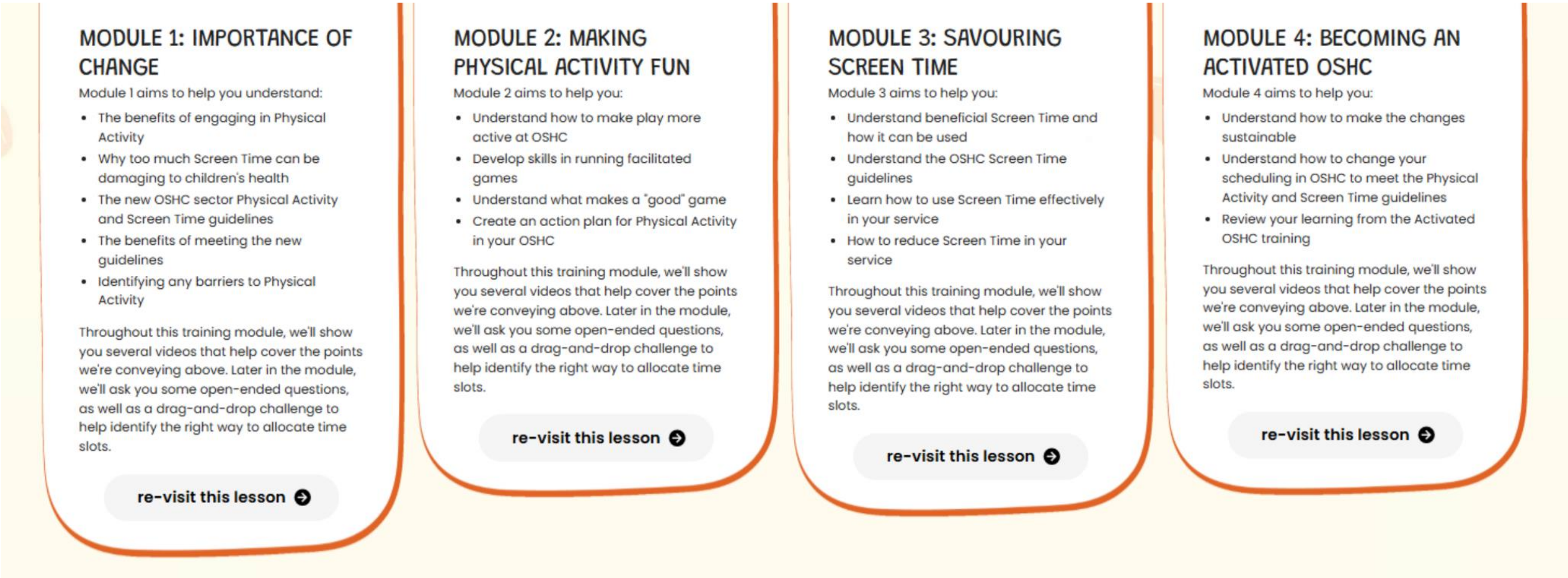

© 2026 Maher C et al. *JAMA Network Open.*

eFigure 2. Activated OSHC Resources Available to OSHC Services

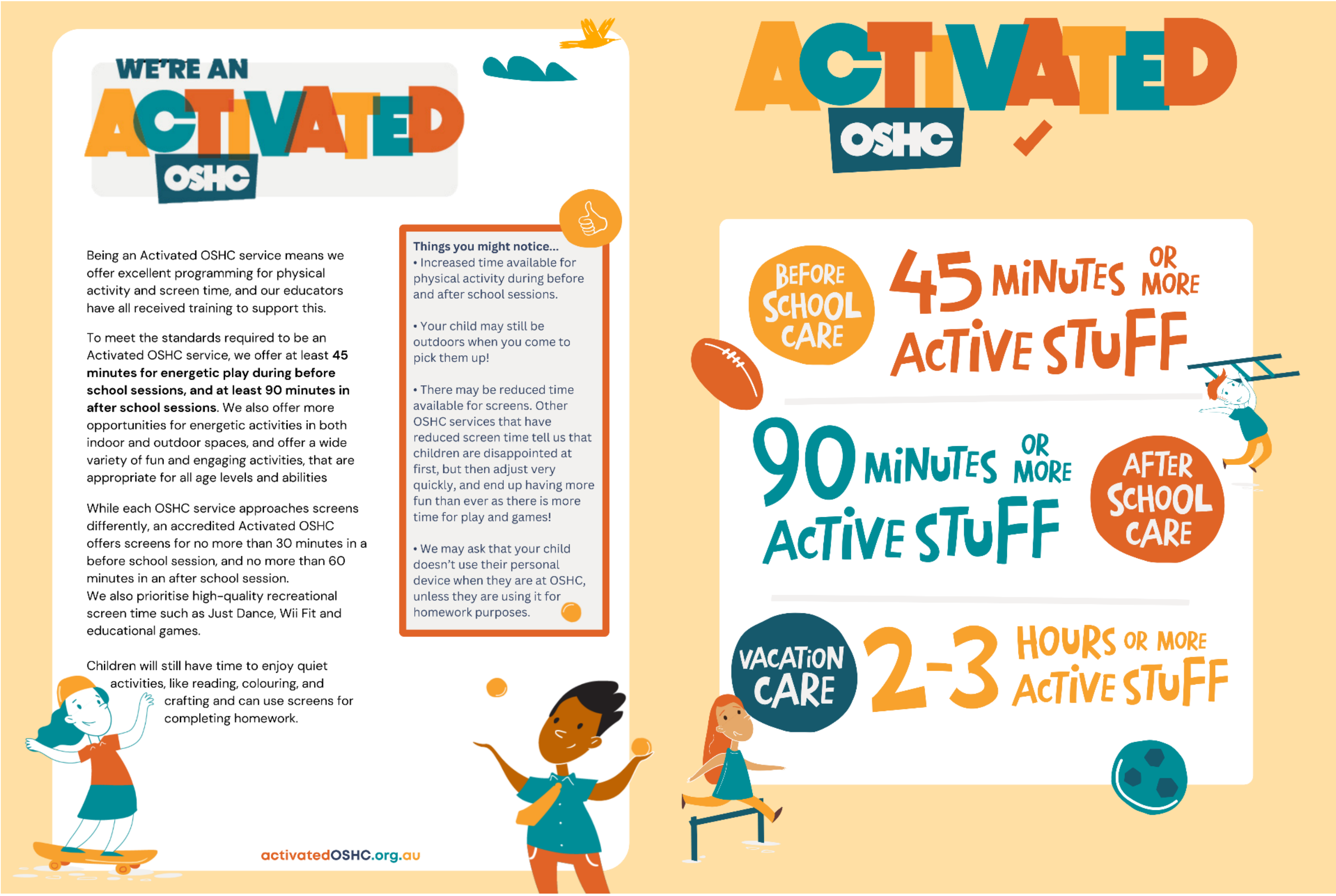

© 2026 Maher C et al. *JAMA Network Open.*

eFigure 3. Out of School Hours Care Services Adherence to OSHC Sector Physical Activity and Screen Time Guidelines at Baseline, 3 Months and 12 Months

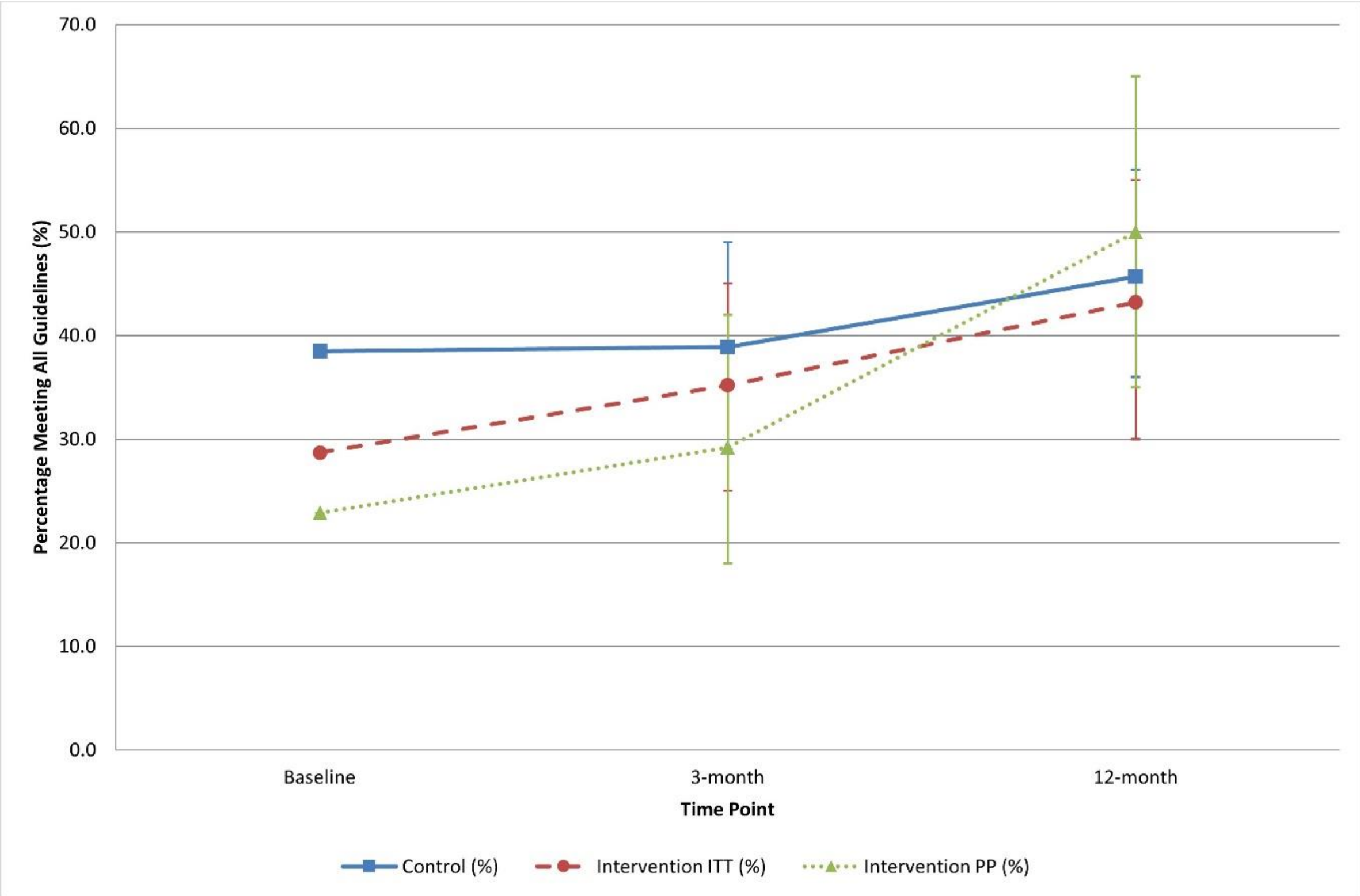

• Control: Solid line (——) with square markers (■) - Control group services

• Intervention (ITT): Dashed line (- - -) with circle markers (●) - Intention-to-treat analysis

• Intervention (PP): Dotted line (· · ·) with triangle markers (▲) - Per-protocol analysis

• Error bars represent 95% confidence intervals

Abbreviations: CI, confidence interval; ITT, intention-to-treat; PP, per-protocol

© 2026 Maher C et al. *JAMA Network Open.*
