# Supplementary material for: A Policy-Based Intervention for Physical Activity and Screen Time in Childcare Outside School Hours: A Cluster Randomized Clinical Trial
Source: JAMA Netw Open. 2026 Oct 5;9(10):e2636741. doi: 10.1001/jamanetworkopen.2026.36741 (PMC13639758; doi:10.1001/jamanetworkopen.2026.36741)
Supplement: Supplementary file 3 [file jamanetwopen-e2636741-s003.pdf]

Data Sharing Statement

Maher. A Policy-Based Intervention for Physical Activity and Screen Time in Childcare Outside School Hours. *JAMA Netw Open*. Published October 05, 2026.
doi:10.1001/jamanetworkopen.2026.36741

Data
Additional Information: Australia New Zealand Clinical Trials Registry (ACTRN12622000393752) - https://www.anzctr.org.au/Trial/Registration/TrialReview.aspx?id=383462&isReview=true
Data available: Yes
Data types: Deidentified participant data, Data dictionary
How to access data: carol.maher@adelaide.edu.au
When available: With publication

Supporting Documents
Document types: None

Additional Information
Who can access the data: Data will be made available to researchers whose proposed use has been approved by the research team, for the purpose of achieving the aims outlined in the approved proposal. Data requests will be considered on an individual basis.
Types of analyses: For a specified purpose outlined in the approved proposal.
Mechanisms of data availability: After approval of a proposal with a signed data access agreement.
